# Controversies in ameloblastoma management: evaluation of decision making, based on a retrospective analysis

**DOI:** 10.4317/medoral.24104

**Published:** 2020-10-09

**Authors:** Andrii Hresko, Olga Burtyn, Leonid Pavlovskiy, Pavlo Snisarevskyi, Julia Lapshyna, Yurii Chepurnyi, Andrii Kopchak, K. Hakki Karagozoglu, Tymour Forouzanfar

**Affiliations:** 1 Centre of maxillofacial surgery and dentistry, Kyiv regional clinical hospital, Kyiv, Ukraine; 2 Head and Neck Oncology Department, National Cancer Institute, Kyiv, Ukraine; 3 Department of Advanced treatment technologies, Kyiv city clinical hospital №12 Kyiv, Ukraine; 4 Pathomorfological department, Kyiv regional clinical hospital, Kyiv, Ukraine; 5 Stomatology department, O.O. Bogomolets National Medical University, Kyiv, Ukraine; 6 Department of Oral and Maxillofacial Surgery/Oral Pathology, VU University Medical Center/Academic Center for Dentistry Amsterdam (ACTA), Amsterdam, The Netherlands

## Abstract

**Background:**

The ameloblastoma management is still challenging to the high recurrence rates and significant morbidity associated with radical treatment. The purpose of this 10-year retrospective study was to analyze the influence of ameloblastoma type and treatment strategy on the long-term outcomes and recurrence rates.

**Material and Methods:**

The retrospective analyses of 64 histologically-confirmed ameloblastoma cases was performed. The possible risk factors for recurrence and the development of complications were estimated statistically.

**Results:**

The treatment strategy applied for this group of patients was the following: thirty-four patients (53.1%) were treated conservatively with enucleation or extended bone curettage. Radical treatment (bone resection) was applied in 30 (46.9%) cases. The follow-up period ranged from 2 to 10 years (mean value 4.28 ± 3,26). General recurrence rate consisted 32.8%. This study did not find significant correlations between clinical or histopathological features of the ameloblastoma and the recurrence rate. The only factor that significantly influence recurrence rate was the treatment strategy (41% in conservative treatment vs 15% in radical treatment, *p*<0.05). Postoperative complications were observed in 42 patients (65.6%) and included face asymmetry and disfigurement (17.2%), temporary paresthesia of the inferior alveolar nerve (IAN) - 23.4%, permanent paresthesia of IAN - 20.3%, paresis of a marginal branch of the facial nerve - 6.3%, infection 12.5%, and swelling - 20.3%. The complication rates, esthetic and functional deficiency were significantly higher in radically treated patients (*p*<0.05)

**Conclusions:**

Our study confirms that higher recurrence rate is associated with conservative treatment for ameloblastoma, while radical treatment leads to an increased number of postoperative complications that affect the patient's quality of life.

** Key words:**Ameloblastoma, ameloblastoma recurrence, odontogenic tumor, oral pathology.

## Introduction

An ameloblastoma, which is a benign but locally aggressive tumor with a high tendency to recur, consists of proliferating odontogenic epithelium lying in a fibrous stroma (1). It is one of the most prevalent odontogenic tumors, accounting for 9-14% of all odontogenic tumors, and constitutes approximately 1% of all oral neoplasms (2,3). The clinical manifestation of ameloblastoma is non-specific and depends on tumor type and localization (4). It can progress to large sizes and cause facial asymmetry, teeth displacement, movability, and malocclusion, as well as pathologic fractures (5,6).

There is still a major controversy concerning which mode of therapy is best based on the clinical presentation or histopathological characteristics of the ameloblastoma (7). The main goals of ameloblastoma treatment are complete surgical removal of the jaw tumor and restoration of masticatory function and facial aesthetics (8). These goals can be achieved via the radical approach – marginal or segmental resection of the affected jaw with immediate or delayed reconstruction, or the conservative method – enucleating and extended curettage (9). In clinical practice, decision making and the choice of the appropriate treatment strategy are still challenging for both the doctor and patient.

The recurrence rates for ameloblastoma are reported to be as high as 15% to 25% after radical treatment and 55% to 90% after conservative treatment (10). Therefore, radical surgery has been recognized as the most effective treatment modality, however, it is associated with a higher rate of complications, leading to patient invalidation, and requires more sophisticated approaches to cosmetic and functional rehabilitation (11,12). On the other hand, recent advances in the understanding of the biological features of ameloblastoma have led to more successful conservative treatments. The latest systemic reviews have demonstrated that for aggressive forms, such as conventional ameloblastoma, radical surgical treatment is more appropriate, while unicystic and peripheral ameloblastomas can be treated conservatively (1,13,14).

The purpose of this retrospective study was to analyze the influence of ameloblastoma type and treatment strategy on the long-term outcomes and recurrence rates over a 10-year period.

## Material and Methods

The retrospective analyses of all pathomorphologically confirmed ameloblastomas treated at the Kyiv regional clinical hospital and National Cancer Institute, Kyiv, Ukraine over the period from 1st January 2009 until 31st December 2018 was performed. During this period of time 88 patients were diagnosed with ameloblastoma. The patients’ files, CT and X-ray data were carefully studied and original histological preparations were revised by experienced pathologist to reconfirm the diagnosis or modify it when necessary. All the recurrent tumors were checked for the signs of malignancy.

The inclusion criteria were histologically-confirmed diagnosis of ameloblastoma and definite surgical treatment performed. The patients without essential clinical information or with signs of malignant transformation were excluded from the study. Out of 88 qualifying ameloblastoma cases, 18 were excluded due to insufficient information in medical records. In 6 cases ameloblastic carcinoma was identified, so we did not account them in further statistic analysis. Among all the cases, 64 patients met the study’s inclusion criteria and were selected for the further analysis.

Tumor types were classified according to the criteria used in the 2017 WHO classification of odontogenic tumors (15). For each patient, the data concerning gender, age, personal history (alcohol and/or drug use, smoking), preoperative diagnosis, tumor location, size, clinical signs and symptoms, radiographic appearance, involvement of the teeth, and surgical management, including reconstruction procedures, histological type, recurrences, and complications, were collected from medical reports, then reviewed and analyzed retrospectively. The localization of the ameloblastoma was categorized according to the system reported by Hong *et al*. and followed by other authors into anterior mandible (cuspid to cuspid), left and right posterior mandibles (premolar and molar), both rami (from the third molar to condyle), an anterior maxilla (cuspid to cuspid), and both posterior maxilla (premolar to pterygoid plates) (16,17). If the tumor involved two or more locations, all locations were included in the database. Treatment was divided into radical and conservative methods, both performed according to the standard protocols. Radical treatment included either a mandibulectomy (marginal, segmental, or hemimandiblectomy) or maxillectomy. Conservative treatment involved enucleation or bone curettage. The bone defects were reconstructed using standard plates or patient-specific implants. For the bone plastics, free fibulae flap or iliac crest bone grafts were used.

For evaluation of the long-term treatment outcome information about results of aesthetic functional and dental rehabilitation, complications and recurrences were collected from clinical examination of the patients and personal interview with a standard questionnaire. The follow-up period ranged from 2 years to 10 years (mean value 4.28 ± 3,26). The research approved by Bioethics Committee of Bogomolets National Medical University, Kyiv, Ukraine (Protocol No. 107). The database with all cases of ameloblastoma was created and analyzed statistically using IBM SPSS Statistics Version 22 (IBM, Armonk, and North Castle, NY, USA). A descriptive analysis of patient characteristics was performed using means and standard deviations for continuous variables and percentage for categorical variables. Comparisons between subgroups were performed using the chi-square test and the Mann–Whitney U test, both with the level of significance set at *p*<0.05.

## Results

Of the 64 patients included in this study, 59.4% were females, and 40.6% males. The patients’ ages ranged from 15 to 73 years. In total, 58 cases of conventional (solid/multicystic) ameloblastoma (90.6%) were diagnosed, whereas unicystic ameloblastomas were observed in 6 (9.4%) cases. There were no cases of extraosseous ameloblastoma in our series. The histological subtypes of the ameloblastomas were: follicular type – 23 (35.9%) cases, plexiform - 4 (6.3%), basal cell - 2 (3.1%), mixed - 6 (9.4%), unicystic - 6 (9.4%), and not specified - 23 (35.9%).

The mandible was affected in 56 (87.5%) patients. In the vast majority of cases, the tumor had arisen in the posterior part of the mandibular body or at the angle region and spread to the ramus and condyle. Eight patients (12.5%) were diagnosed with maxillary ameloblastoma, of which two were located in the anterior maxilla, five were in the posterior, and one affected both anterior and posterior maxilla. A unilocular radiographic appearance was present in 32 cases (50%), and in 32 (50%) cases, the appearance was multilocular. We found a total of 39 (60.9%) patients who had complaints, including pain (37.5%), swelling (35.9%), temporary paresthesia of the inferior alveolar nerve (IAN) (20.3%), and face asymmetry (1.6%). In 25 cases (39.1%), patients had no complaints; in these cases, the tumors were accidentally found during dental treatment or X-ray examination of the jaws. Teeth were involved with the tumor in 34 cases (53.1%).

All patients underwent biopsies to confirm their diagnoses. Thirty-four (53.1%) ameloblastoma cases were treated conservatively with enucleation or extended bone curettage. Radical treatment (bone resection) was applied in 30 (46.9%) cases. Recurrences were observed in 21 cases (32.8%), 18 (28.1%) of which occurred after primary conservative surgery, while three (4.7%) followed ablative surgery. The mean period between surgery and clinical or radiological manifestation of the tumor recurrence was 3.61 ± 2.37 (range from 1 to 7 years). The recurrence was noted during first two years follow up in 42.9% of cases, between two and five years – 33.3%, and only 23.8% were diagnose after 5 years follow up. Postoperative complications were observed in 42 patients (65.6%) and included: face asymmetry and disFigurement (17.2%), temporary paresthesia of the inferior alveolar nerve IAN (23.4%), permanent paresthesia of the IAN (20.3%), paresis of the marginal branch of the facial nerve (6.3%), infection (12.5%), and swelling (20.3%). In this study, we documented a low rate of dental rehabilitation after ameloblastoma treatment. Only 34.4 % of patients received prosthetic rehabilitation, mostly through the application of removable dentures (Table 1).

The patients, analyzed in our study underwent 119 operations, in general, and 1.9±1.1 (mean± s.d.), on average. The mean number of operations for patients treated conservatively was 1.2 ± 0.5 (ranged from 1 to 3) interventions per person, which were mainly bone curettage or enucleation. At the same time, the mean number of surgeries undergone by radically treated patients was 2.6 ± 1.1 per person (ranged from 1 to 7). A large variety of different surgeries was documented for this group of patients and included biopsies, resections with or without bone grafting procedures, and surgical revisions due to local infections of bone grafts, failed fixation systems, secondary reconstructions, and so forth.

**Table 1:**
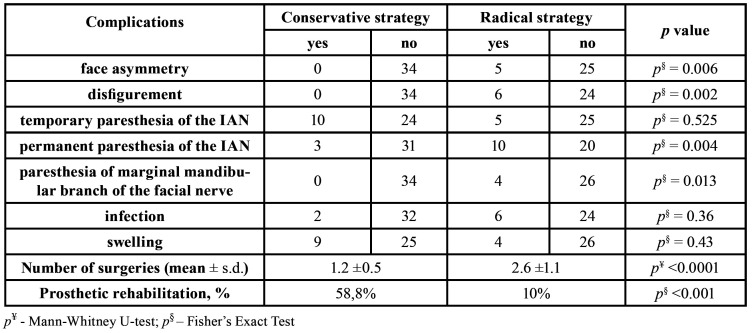
Correlations between the long-term outcomes and treatment strategies.

## Discussion

In a number of retrospective international studies on ameloblastoma epidemiology, its clinico-pathological patterns, and treatment strategies were quite significant. The largest dataset was presented in a systemic review by Reichart *et al*. and included 3677 patients with this tumor type from different countries and regions (6). According to that study, ameloblastoma most commonly presents as hard, bony swelling over the affected region (mandible in 80% of cases) at an average age of 36 years with equal sex distribution. At the same time, there are some differences in ameloblastoma demographics reported for different regions as well as ethnic and racial groups. For example, the peak incidence of ameloblastoma in Asia fell in the third decade of life, while it fell in the fifth decade of life in the USA (16). The present study demonstrated that ameloblastoma patterns in Kyiv, Ukraine are close to those reported for the Caucasian populations in other countries (Europe and the USA). The mean age of the patients was 42.95 ± 15.8 years, nearly identical to those in studies conducted by Oomens *et al*. [44.1] (18).

The predominant type of tumor in the present study was conventional ameloblastoma (90.6%), followed by unicystic ameloblastoma (9.4%). The same proportion was reported in the study of Filizzola *et al*., which reported 81% conventional and 13% unicystic ameloblastomas and other studies (19,20). We did not observe any cases of extraosseous ameloblastoma in our group of patients. The explanation for this may be that this type of tumor is quite rare, with a prevalence rate from 0.5% to 9.3%. The other possible reason is that it can be easily misdiagnosed as other common, benign oral tumors or treated in outpatient clinics. The distribution of the tumor subtypes and histologic patterns in our study were similar to what has been reported in the literature. The follicular pattern had a strong predominance, accounting for 31% of cases. (18,21). Furthermore, 87.5% of the ameloblastoma cases were encountered in the mandible, which is consistent with reports by Krishnapillai *et al*. (91.8%), Masthan *et al*. (80%), Dhanuthai *et al*. (84.3%), and Becelli *et al*. (80%) (3,22-24). Most of the cases were located in the posterior mandible (molar, angle, and ramus regions). However, in our study, none of these clinico-pathological factors influenced the recurrence rate significantly (*p*=0.37) (Table 2). Milman *et al*. also observe that there was no significant association between histologic pattern and tumor recurrence (*p* = 0.48) (25). These findings differ from the results obtained by Richart *et al*. and Hong *et al*., who reported significant correlations between histological type and recurrence rate (6,16). The explanation for this may be that this study is limited by its small sample size, and therefore the results should be interpreted with caution.

The treatment of ameloblastoma can be performed conservatively (by enucleation or curettage) or radically (marginal, segmental resection, hemimandibulectomy, or maxillectomy) (25). Ameloblastoma has a much higher recurrence rate then other benign jaw tumors (26,27). Late diagnoses (39.1% of cases were diagnosed accidentally in our study) and the spread of the lesion to more than one location (46.9% of our cases) lead to poor prognoses for aesthetic and functional rehabilitation. Therefore, many multicenter studies have advocated for more aggressive, radical methods of ameloblastoma management (23,28). According to the literature, the treatment strategy is the main factor that influences the recurrence rate, as well as the risk of postoperative complications (29). In our study, 34 (53.1%) patients were treated conservatively, a much higher percentage than in Hatada *et al*. (35.7%) but similar to that in Ruslin *et al*. (62.8%) (17,28). The number of recurrences in patients who were treated conservatively in our study was 28.1% vs 4.7% in patients who underwent radical treatment (the difference was statistically significant). However, the total recurrent rate in our study was 32.8%, much higher than the percentages reported by Antonoglou *et al*. (15.2 %) and Krishnapillai *et al*. (12.4%) (22,30). At the same time, our study confirmed that the radical treatment strategy is associated with a significantly higher risk of post-surgical complications, a lower rate of prosthetic rehabilitation, and the necessity of numerous surgical interventions compared to the patients treated conservatively.

**Table 2:**
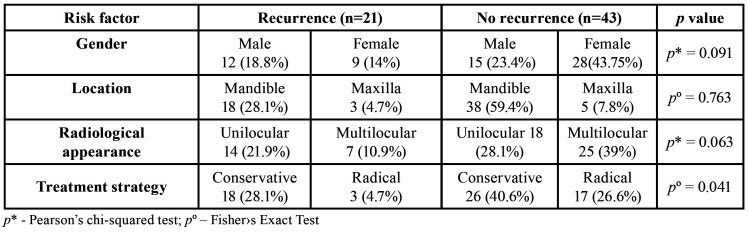
Correlations between the possible risk factors and recurrence rates

These findings correlate with the results of Hendra *et al*. and Hasegawa *et al*., who suggest that the radical treatment strategy has severe consequences for the patient and reduces their quality of life (14,31). In developing countries with limited resources devoted to the health care system, these factors significantly influence decision making: conservative methods are associated with a higher risk of recurrence but lower risks of other types of complications and fewer surgical interventions for esthetic and functional rehabilitation. Consequently, this strategy receives a better response from patients. The retrospective results obtained in our study confirm the necessity for defining less invasive approaches to ameloblastoma elimination and ensuring a high quality of life for patients.

The present study has several limitations. It was limited to only 2 centers and the number of patients was relatively small to analyze the true incidence and behavior of the rare histological subtypes like extraosseous ameloblastoma. Also, we didn’t analyze the influence of histological pattern of unicystic ameloblastoma on the treatment outcomes due to the very small number of these types of tumors. Because this study was retrospective, the analysis may include an information bias regarding both clinical and morphological data. The large number of cases was excluded from the study due to the incomplete reporting and lack of important information in medical records. The minimal follow up period was 2 years, and the recurrences may develop and manifest at later terms (up to 7 years according to our data). However, the data obtained in the study is quite comprehensive and comparable with the reports from other studies. It also provides the new information which can be beneficial for better understanding of the tumor epidemiology and development of the treatment strategies.

## Conclusions

Our study confirms that the treatment strategy is the main factor that influence the recurrence rates and risk for complication development. Higher recurrence rate is associated with conservative treatment for ameloblastoma (28.1 % vs 4.7 % in radically treated patients, *p*<0.05), while radical treatment leads to an increased number of postoperative complications that affect the patient's quality of life.
